# Integrated transcriptome catalogue and organ-specific profiling of gene expression in fertile garlic (*Allium sativum* L.)

**DOI:** 10.1186/s12864-015-1212-2

**Published:** 2015-01-22

**Authors:** Rina Kamenetsky, Adi Faigenboim, Einat Shemesh Mayer, Tomer Ben Michael, Chen Gershberg, Sagie Kimhi, Itzhak Esquira, Sarit Rohkin Shalom, Dani Eshel, Haim D Rabinowitch, Amir Sherman

**Affiliations:** Institute of Plant Sciences, ARO, The Volcani Center, Bet Dagan, Israel; Robert H. Smith Faculty of Agricultural, Food, and Environmental Quality Sciences, The Hebrew University of Jerusalem, Jerusalem, Israel; Classeed Ltd., Gibraltar, British; Institute of Postharvest and The Food Sciences, ARO, The Volcani Center, Bet Dagan, Israel

**Keywords:** Assembly, Flowering, Breeding, Sulfur metabolism, Garlic virus

## Abstract

**Background:**

Garlic is cultivated and consumed worldwide as a popular condiment and green vegetable with medicinal and neutraceutical properties. Garlic cultivars do not produce seeds, and therefore, this plant has not been the subject of either classical breeding or genetic studies. However, recent achievements in fertility restoration in a number of genotypes have led to flowering and seed production, thus enabling genetic studies and breeding in garlic.

**Results:**

A transcriptome catalogue of fertile garlic was produced from multiplexed gene libraries, using RNA collected from various plant organs, including inflorescences and flowers. Over 32 million 250-bp paired-end reads were assembled into an extensive transcriptome of 240,000 contigs. An abundant transcriptome assembled separately from 102,000 highly expressed contigs was annotated and analyzed for gene ontology and metabolic pathways. Organ-specific analysis showed significant variation of gene expression between plant organs, with the highest number of specific reads in inflorescences and flowers. Analysis of the enriched biological processes and molecular functions revealed characteristic patterns for stress response, flower development and photosynthetic activity. Orthologues of key flowering genes were differentially expressed, not only in reproductive tissues, but also in leaves and bulbs, suggesting their role in flower-signal transduction and the bulbing process. More than 100 variants and isoforms of enzymes involved in organosulfur metabolism were differentially expressed and had organ-specific patterns. In addition to plant genes, viral RNA of at least four garlic viruses was detected, mostly in the roots and cloves, whereas only 1–4% of the reads were found in the foliage leaves.

**Conclusions:**

The *de novo* transcriptome of fertile garlic represents a new resource for research and breeding of this important crop, as well as for the development of effective molecular markers for useful traits, including fertility and seed production, resistance to pests and neutraceutical characteristics.

**Electronic supplementary material:**

The online version of this article (doi:10.1186/s12864-015-1212-2) contains supplementary material, which is available to authorized users.

## Background

Garlic (*Allium sativum* L.), the second most important *Allium* crop after the bulb onion, is cultivated worldwide and consumed by almost every culture as a popular condiment and green vegetable. It is also known for its medicinal and neutraceutical properties, with a large spectrum of antibacterial and anti-inflammatory activity [1-3]. None of the known commercial garlic cultivars and landraces produce fertile flowers or seeds. Therefore, garlic is not propagated sexually, and neither classical breeding nor genetic studies are currently employed with this plant. Nevertheless, over the past 20 years, fertility restoration has been achieved in a number of garlic genotypes, mostly landraces from Central Asia [4-8], and physiological studies have led to flowering induction and seed production by environmental manipulation [8,9]. Self- and cross-pollination within and between garlic genotypes has become a reality, thus enabling the initiation of genetic studies and classical breeding in garlic [2,10-14].

Garlic generates the highest known concentration of organosulfur compounds among *Allium* species. These are responsible for both garlic’s pungent odor and many of its medicinal benefits. The levels of the flavor precursors, non-protein cysteine and glutathione derivatives, account for 1–5% of the garlic's dry weight [15], indicating the major importance of organosulfur biosynthetic activity within the plants' tissues. These compounds serve two roles in the life cycle of the plant: pest deterrence and the storage and transport of carbon, nitrogen, and sulfur [15]. The major garlic flavor precursor is sulfur-alk(en)yl cysteine sulfoxide (alliin). This chemical is broken down by alliinase to pyruvate, ammonia and thiosulfinate allicin, which are the sources of the typical aroma and proposed health-beneficial properties of garlic [3,16,17].

Like many perennial monocots, edible *Allium* species have very large genome sizes of 10–20 Gbp [18]. The diploid garlic (2n = 2x = 16) nuclear genome is estimated at 15.9 Gbp, 32 times larger than the genome of rice [19]. Therefore, full sequencing of the garlic genome is a challenging task, but transcriptome assembly using next generation sequencing might be efficiently employed for the generation of functional genomic data. This approach has been successfully applied to perennial crops, e.g., peony [20], pomegranate [21], orchid [22] and *Arundo donax* [23], as well as to the bulbous monocots *Tulipa* and *Lilium* [24,25] and onion [26]. In garlic, the first transcriptome analysis based on RNA from the renewal buds resulted in *de novo* assembly of 128,000 unigenes which were annotated and analyzed with respect to gene ontology (GO) and metabolic pathways [27]. In addition, 352 differentially expressed transcript-derived fragments showed differential expression in the leaf, meristematic and flower tissues [28].

Since only a few garlic genotypes are able to produce flowers and viable seeds, getting this plant to flower is still a challenge, and genetic regulation of the reproductive process is of special interest [2]. Recent physiological studies have facilitated flowering induction and fertility restoration by environmental manipulations in a variety of garlic genotypes [9,11,13], and our research group currently maintains numerous genotypes of fertile seed-producing garlic genotypes. A further and deeper understanding of the genetic make-up of garlic reproductive traits is required for both improved genetic and physiological knowledge and exploitation of the genetic potential of this important crop.

In this report, we describe the *de novo* assembly of a transcriptome catalogue for fertile garlic, and provide a few examples of its analysis. This is the first reported garlic transcriptome representing genes expressed in vegetative and reproductive organs, which were sampled and analyzed throughout plant development, from clove sprouting to flowering and bulbing. The results provide a platform for further research and breeding of this important crop.

## Results and discussion

### Transcriptome sequencing and annotation

To establish a transcriptome catalogue for fertile garlic, we used MiSeq pair-end technology, which enabled us to create an assembly of longer contigs. While the long-read protocol is considerably more expensive than short-read sequencing, important benefits include a lower mapping bias and reduced ambiguity in assigning reads to genomic elements, such as mRNA transcripts [29]. Six cDNA libraries from six garlic organs yielded approximately 32.6 million long 250-bp paired-end reads (Table 1). Quality trimming and filtration resulted in 28.4 million cleaned reads that were assembled using Trinity, and generated 239,116 contigs for the "extensive transcriptome catalogue". The average contig length was 715 bp; half of these (N50) were at least 1,088 bp long. The elimination of contigs with fewer than 10 total mapped reads resulted in a further reduction, resulting in a total of 102,042 contigs, assembled separately into an “abundant transcriptome catalogue”. Clustering the sequences with 95% identity revealed a redundancy of less than 10% in both the extensive and abundant transcriptomes.

**Table 1:** **Statistical summary of extensive and abundant transcriptome catalogues of the fertile garlic**

	**Total in plant**	**Root**	**Basal plate**	**Leaf**	**Clove**	**Inflorescence**	**Flowers**
Read pairs	32,607,803	5,460,636	5,654,667	4,228,289	5,539,119	6,969,526	4,755,566
Cleaned read pairs	28,430,270	4,737,985	5,007,820	3,729,942	4,777,731	6,013,904	4,162,888
Extensive transcriptome	239,116	104,937	111,289	85,423	144,405	236,946	203,781
Unique components	183,328	75,592	74,221	62,676	92,782	129,965	122,490
Abundant Transcriptome*	102,042	30,390	33,155	25,109	39,119	62,678	53,066

cDNA libraries were sampled from six vegetative and reproductive organs.

*Contigs computed with count per sample >10.

**Figure 1:**
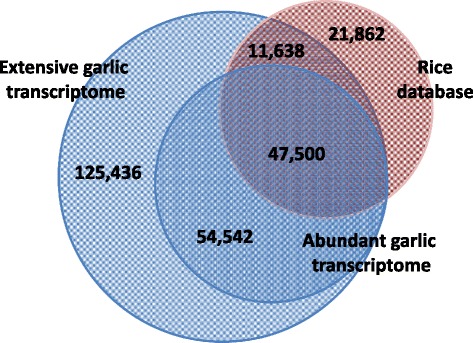
**Venn diagram of the distribution and similarity of sequences in extensive and abundant transcriptome catalogues of garlic in comparison with rice protein database (** **www.phytozome.org** **).**

Both transcriptome catalogues of garlic were compared with the database of *Oryza sativa*—the most sequenced and annotated monocot species (Figure 1). BLASTX search against the rice database resulted in at least one significant hit for 59,138 contigs of the extensive garlic transcriptome (24.7%); 47,500 of those contigs represented 46.5% of the contigs of the abundant transcriptome. In a reciprocal TBLASTN search of the rice genes in the extensive garlic transcriptome, at least one significant hit was found for 44,476 rice sequences, i.e., 67% of the rice sequences were found in garlic (Figure 1).

Comparison of the abundant garlic transcriptome with public databases revealed various levels of similarity with the model species *Arabidopsis* and *Oryza*, as well as other bulbous geophytes (Table 2). Databases of *Arabidopsis*, rice and nrNCBI showed a general similarity of 45–47% with our data. In addition, the nrNCBI protein database was used to search for the five top hits. Among the 47,509 contigs annotated in the abundant transcriptome, 27% show high similarity to *Oryza sativa*, 8.4% to *Hordeum*, 5.52% to *Triticum*, and 8% to *Arabidopsis* (search results from April 2014). The main reason for such similarity might be higher representation of the genomic resources for these species. There were abundant transcriptome matches with 463 published genes from 10 *Allium* species and 458 genes from 33 geophyte species that were closely or remotely related to garlic (e.g., *Lilium, Narcissus, Tulipa*, and *Agapanthus*). Of the contigs obtained here, 44 corresponded with at least 4 known garlic viruses.

**Table 2:** **Global sequence similarity of garlic abundant transcriptome to transcriptome databases for model and related species**

**Reference**	**Reference database**	**Number of reference sequences**	**Percent of hits in reference database**
nrNCBI	http://www.ncbi.nlm.nih.gov	~169.000.000	46.6
*Arabidopsis*	http://www.arabidopsis.org	41,671	45.8
*Oryza sativa*	www.phytozome.org	66,338	46.6
*Allium cepa*	http://www.ncbi.nlm.nih.gov PRJNA175446	26,995	57.8
*Allium cepa*	http://www.ncbi.nlm.nih.gov PRJNA175449	33,162	51.3
*Allium sativum* (renewal bud)	http://www.ncbi.nlm.nih.gov PRJNA158177	79,143	77.8
*Tulipa* (leaves)	[24,25]	52172	44.5*
*Lilium* (leaves)	[24,25]	79980	45.3*

*Based on 25,109 sequences specific to garlic leaves.

Although 78% of the sequences were homologous with the previously reported transcriptome of garlic renewal bud [27], reads of our transcriptome were much longer (N50 of 1,088 bp) than those of this previous report (based only on 90 bp paired-end reads), and therefore represented larger portions of the full genes. In addition, BLASTN analysis identified 51–58% similarity of the reference transcriptome with onion accessions [26]. It is interesting to note that the similarity between the abundant catalogue and the published sequences of bulbous monocots *Lilium* and *Tulipa* [24,25] was rather low—only 21.3 and 20.6%, respectively. However, taking into account that reports for *Lilium* and *Tulipa* include only the leaf transcriptome, we repeated the BLASTN analysis with the 25,109 contigs specific to garlic leaves. In this case, much higher match was obtained, 45.3 and 44.5% for *Lilium* and *Tulipa*, respectively. In comparison, the resemblance between rice and the annotated transcriptomes of *Lilium* and *Tulipa* reaches 49% and 30%, respectively [24]. This clearly demonstrates that comparative analyses between transcriptomes that include samples from various organs at different stages of plant development provide more reliable results. Transcriptome analysis from only one plant organ or tissue does not provide a full transcript catalogue, even though it can serve numerous specific genetic and breeding objectives [25].

Comparative phylogeny of monocots has suggested that the divergence between Liliales (e.g., *Tulipa* and *Lilium*) and Asparagales (*Allium*) occurred between 120 and 133 million years ago, while divergence between Poales (*Oryza*) and Asparagales took place later, between 98 and 116 million years ago [30,31]. Although Asparagales and Liliales have evolved many traits in parallel and it is difficult to separate them morphologically, gene-sequencing analysis supports these two orders as two separate monophyletic groups [32]. Rice might be phylogenetically closer to Asparagales than to Liliales, and therefore, only a relatively low number of shared possible orthologous groups are identified between rice, garlic, *Tulipa* and *Lilium*. On the other hand, the low ratio of similarity demonstrates a potential source of unidentified specific genes that need to be annotated in bulbous monocots. It is also possible that genes from bulbous monocots deviate at the sequence level from the allied genes existing in the databases, or that some of the transcripts in the published transcriptomes were not long enough to find a significant similarity with annotated genes.

### Organ-specific expression analysis

**Figure 2:**
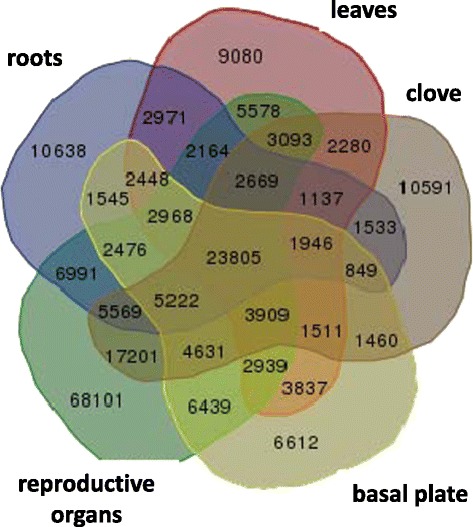
**Common and specific contigs found in the extensive transcriptome catalogue of the various organs of fertile garlic.** Note the high number of specific contigs in the reproductive tissues. Samples of the reproductive organs (young inflorescences and flowers at various stages of development) are unified for this analysis.

Analysis of the extensive transcriptome showed that most contigs are shared between plant organs, but only 13.7% (23,805) are common to all studied organs (Figure 2). On the other hand, numerous contigs were organ-specific, i.e., to the roots (10,638), leaves (9,080), basal plate (6,612) and clove tissue (10,591). Similarly, large differences in gene-expression patterns between different tissues, especially between root and aerial organs, have been reported for other plants, e.g. *Arabidopsis* [33] and *Glycine max* [34]. In rice, 69% of the expressed transcripts show significantly variable expression levels among tissues/organs [35]. In our study, the number of specific reads was exceptionally high in the reproductive organs—68,101 transcripts expressed only in inflorescences and flowers, representing 28.5% of the extensive transcriptome—thus confirming that the signaling and transcriptional control of reproductive development involves a number of regulatory mechanisms and a large number of genes. Similarly, 25,062 pollen-preferential transcripts are expressed in rice pollen [36], and 1,145 soybean genes are flower-specific [34]. Detailed transcriptome comparisons between different species may reveal evolutionarily conserved genes and molecular mechanisms in plant reproduction processes. Such comparisons require comprehensive transcriptome datasets of tissues and developmental stages, and precise annotations of orthologous genes between species [37].

**Figure 3:**
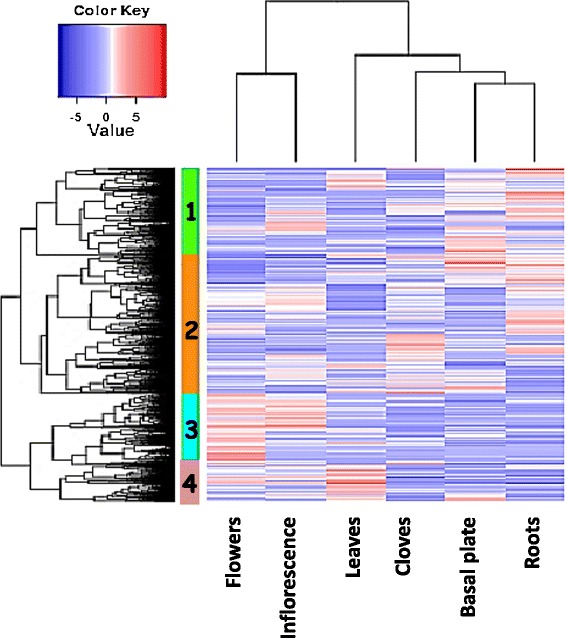
**Hierarchical cluster analysis of gene-expression patterns in six vegetative and reproductive organs of garlic.** The heat map shows the relative expression levels of each contig (rows) in each sample (columns). Four identified gene clusters (shown in the left tree) are differentially expressed in one or more organs. Organs are clustered to reproductive and vegetative, with closer proximity between the roots and basal plates (upper tree). Expression values (FPKM) are log2-transformed and then median-centered by transcript.

Global gene-expression profiling of the abundant transcriptome revealed differential patterns between the vegetative and reproductive organs (Figure 3). The vegetative organs could be clustered into two defined groups, with the underground organs—roots and basal plate—sharing both close proximity and many expressed sequences. In bulbous geophytes, the basal plate (the compressed actual stem) is morphologically associated with the roots, leaves and renewal buds [38]. When the reproductive stage begins, an apical meristem forms the floral scape and inflorescence. Flower differentiation in garlic is affected by endogenous and environmental signals. However, it is not clear whether florogenesis is regulated by direct signal transduction from the vegetative organs, or is only supported by photosynthetic products and storage materials from foliage leaves and the underground bulb [9,13].

Hierarchical cluster analysis of gene-expression patterns revealed four significantly different groups of contigs (Figure 3). Alterations in gene expression provided information on organography of the expression patterns.

Cluster #1 (highlighted in green in Figure 3) consisted of 2,439 transcripts, enriched mainly in vegetative organs. Employing GO enrichment analysis in this cluster revealed gene-expression patterns reflecting intense metabolic activity, transport, and regulation of the stress response (Additional file 1: Figure S1a). Biological processes related to responses to external stimuli, such as inorganic and organic substances, and to various types of abiotic stress, such as osmotic, oxidative or salt stress, were prominent in the roots and basal plate. Regulation of cellular response to stress in the roots might be induced by endogenous stimuli, by interactions with symbionts and pathogens, or by wounding. The main metabolic functions of cluster #1 included antioxidant activities, especially oxidoreductase, reductase, and carbon-sulfur lyase activities. In plants, the deleterious effect of reactive oxygen species (ROS) accounts for many of the stress conditions and abiotic damage. To protect against injury by free radicals, living organisms have developed a battery of antioxidant defenses, such as the enzymes superoxide dismutase (SOD), catalase and glutathione peroxidase [39]. In addition, small molecules, including glutathione and other organosulfur compounds, play a significant role in the defense against ROS effects. It is therefore suggested that roots might play a substantial role in stress resistance and antioxidant defense in garlic plants.

Cluster #2 (highlighted in orange in Figure 3) contained 3,878 contigs, with a slightly higher ratio of enriched genes in the cloves and roots. However, only a small number of the genes in this cluster were organ-specific. Molecular functions, analyzed by the GO test tool, showed enrichment in GO terms involved in catalytic activity, mainly related to carbohydrate transferases. These findings certainly support the concept of carbohydrate accumulation in the storage organs during growth and dormancy induction in garlic [2,40] and other geophytes [41]. On the other hand, analysis of cluster #2 clearly showed over-representation of genes involved in plant–pathogen interactions, including processes related to viral genome replication and symbiosis between viruses and the garlic plant (Additional file 1: Figure S1b).

Cluster #3 (highlighted in blue in Figure 3) contained 2,007 contigs. Numerous genes, mostly specific to the reproductive process, were abundant (Figure 2, Additional file 2: Figure S2a). The major processes encoded by these genes in the reproductive tissues involved floral morphogenesis, development of male and female reproductive organs, cell division, and gametophyte development. At least 100 GO terms were significantly enriched in the reproductive organs. Nevertheless, GO analyses showed a variety of biological processes, including DNA replication and modification, regulation of RNA synthesis, cell cycle, cellular component organization activity and more (Additional file 2: Figure S2a). At the cellular level, these processes were associated with cytoskeleton and microtubule activities and chromosomes (data not shown). GO terms of flower morphogenesis, pollen formation, cell cycle and organelle organization were notable (Additional file 2: Figure S2a). This variety might be justified by the wide range of developmental stages sampled: reproductive organ samples were collected from very young inflorescences with undifferentiated flower primordia, through flower buds with developing anthers and gynoecia, to fully developed flowers at anthesis.

Cluster #4 (highlighted in pink in Figure 3) was relatively small, consisting of 1,011 contigs with significant representation in the foliage leaves. Carbohydrate metabolic activities and photosynthetic processes were notable (Additional file 2: Figure S2b). In addition, the metabolism and transport of sulfur, phosphorus, and nitrogen were prominent. Genes associated with synthesis of lipid, sugar and aromatic compounds were over-represented, and expression related to metabolic activity was high. In terms of cellular localization, the processes occurred in most organelles, as well as in membranes and intracellular spaces.

Of the 12 most abundant proteins in the abundant transcript catalogue, 5 matched known sequences from onion and garlic, and another 4 were highly similar to proteins reported from *Medicago, Vitis* and *Nelumbo*. Three proteins with highest expression in the leaves and flowers were unidentified (Additional file 3: Table S1).

To date, the identification of genes controlling garlic development or metabolic pathways has been based on a restricted number of gene orthologues [11,14,28]. Establishment of the garlic transcriptome allows for large-scale identification of genes showing tissue-specific expression.

### Orthologues of key flowering genes and their expression in garlic organs

Orthologues of several key flowering genes (Table 3) were found in the transcriptome by matching with known gene sequences from *Arabidopsis*, *Oryza* and *Allium cepa* databases (Additional file 4: Table S2). In model plants, the floral-induction pathways have been shown to be integrated into a flowering network, which contains several steps [42,43]. Thus, in *Arabidopsis,* these pathways involve an array of transcription factors [e.g., *CONSTANS (CO)*, *FLOWERING LOCUS C (FLC*), *SUPPRESSOR OF OVEREXPRESSION OF CONSTANS1 (SOC1*), *LEAFY (LFY*), *APETALA1 (AP1*)], regulators of chromatin structure [*VERNALISATION2* (*VRN2*)], the putative kinase inhibitor *FT*, and many other genes [44,45]. The key genes controlling flowering in *Arabidopsis* are conserved in other species. For instance, in onion, some of these genes are involved not only in the flowering process, but also in the photoperiodic control of bulb initiation [46,47]. Our analysis of gene expression in various organs of garlic demonstrated that orthologues of flowering genes can be involved in numerous biological processes, including flower initiation, stem elongation and bulbing.

**Table 3:** **Orthologues of key flowering genes and their relative expression in garlic vegetative and reproductive organs**

**Flowering genes**	**Comp number**	**Relative expression in different organs**					
		**Roots**	**Basal plate**	**Cloves**	**Leaves**	**Inflorescence**	**Flowers**
*FLOWERING LOCUS T (FT)*	comp149377	0	0	0	0	1.11	3.21
CONSTANS (CO)*	comp45555	0.72	5.99	12.59	5.49	6.87	5.12
*SUPPRESSOR OF OVEREXPRESSION OF CONSTANS1* (*SOC1*)*	comp151688	0	0	0	1.26	1.13	0
*LEAFY (LFY)*	comp44757	1.61	0	0.77	0	25.35	1.65
*APETALA1 (AP1)*	comp38587	2.52	62.14	0	50.43	64.89	5.56
APETALA2 (AP2)*	comp46193	3.15	1.45	8.64	0.98	0.54	22.46
*APETALA3 (AP3)*	comp35626	0	0	0.57	0	40.93	245.92
*PISTILLATA (PI)*	comp44196	0.56	0.55	0.27	0	2.58	143.66
SEPALLATA1 (SEP1)*	comp56369	0	0.93	0	0	86.97	13.48
*SEPALLATA3 (SEP3)*	comp42485	0.52	0	0	0	26.16	263.92
AGAMOUS (AG)*	comp59363	0.9	0	0	0	4.83	185.44

Sequences of known flowering genes from public databases were matched with the garlic abundant transcriptome. The expression level (FPKM) was calculated using the expectation maximization method, and TMM normalization (trimmed mean of M-value normalization method) was applied (see Materials and Methods).

*Numerous sequences with significant similarity were identified.

**Figure 4:**
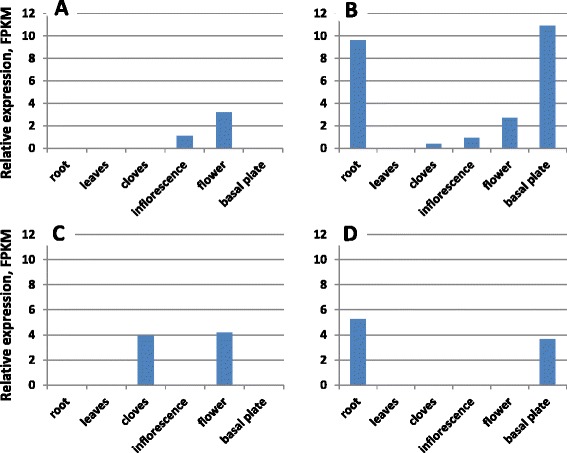
**Differential expression of four** ***FT*** **orthologues in vegetative and reproductive organs of fertile garlic: (A) comp149377_c0_seq1, (B) comp32165_c0_seq1, (C) comp82533_c0_seq1, (D) comp4812_c0_seq1.**

Numerous isoforms identified for some of the key flowering genes, e.g., *FT, AP2, SEP1* and *AG,* varied in their expression in garlic organs. As an example, at least four sequences of *FT* orthologues were differentially expressed, with significant over-representation in inflorescences and flowers (Figure 4 a,b,c), roots and basal plate (Figure 4 b,d), or cloves (Figure 4 c). *FT* homologues are involved in a range of plant developmental processes. In perennial tomato, the orthologue *SFT* has been shown to regulate diverse growth processes, such as the flowering, growth and termination cycles typical of perennial plants, leaf maturation, growth of stems, and the formation of abscission zones [48]. *FT* homologues are involved in leaf development and flower induction in *Narcissus* [49] and tuberization in potato [50]. In onion, at least four different *FT* genes regulate flowering and bulb formation. Flowering is promoted by vernalization and correlates with upregulation of *AcFT2*, whereas bulb formation is regulated by two antagonistic *FT-*like genes [47]. *FT* orthologues identified in the garlic transcriptome demonstrate strong homology to the onion *FT-*like genes and might similarly regulate developmental processes in garlic.

Although garlic genetics is still in its infancy, we believe that new transcriptome data will significantly accelerate research into the genetic regulation of flowering, seed development, dormancy induction and the bulbing process in this important crop. Mining for molecular markers for flowering and fertility is of special interest, since their development will certainly advance our progress toward hybridization and seed propagation in garlic.

### GO classification in tissue-specific sulfur metabolism

Garlic has a high content of organosulfur compounds, but little information is currently available on the molecular regulation of sulfur biosynthesis in this plant [3]. Biosynthetic pathways of alliin precursors involve alk(en)ylation of the amino acid cysteine in glutathione, (thio)alk(en)ylation of cysteine or a precursor such as O-acetylserine. The regulatory complex of cysteine synthase is formed by the enzymes O-acetylserine sulfhydrylase (acetylserine(thiol)-lyase (OASS) and serine acetyltransferase (SAT). These enzymes are at the branch point of the sulfur-, carbon-, and nitrogen-assimilation pathways [51]. In garlic, cysteine and glutathione metabolism occurs in parallel, and variation in metabolic routes depends on the physiological state of the tissue [16,17].

**Figure 5:**
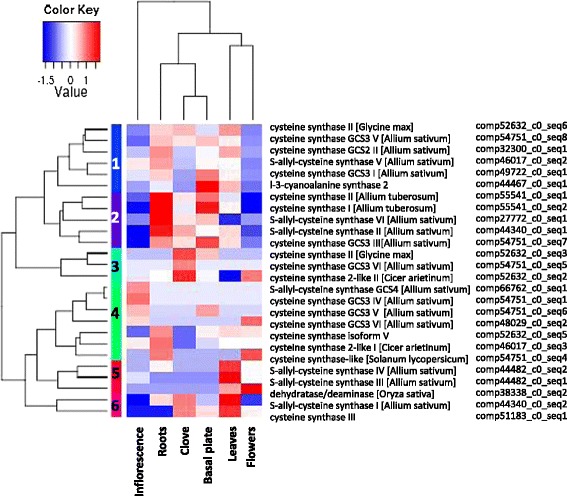
**Hierarchical cluster analysis of gene-expression patterns of 26 annotated variants and isoforms of O-acetylserine(thiol)-lyase (OASS) in six vegetative and reproductive organs of garlic.** The heat map shows the relative expression levels of each variant (rows) in each organ (columns). Expression values (FPKM) are log2-transformed and then median-centered by variant.

Using *de novo* assembly of the garlic transcriptome, we distinguished about 100 variants and isoforms of nine enzymes involved in sulfur metabolism in different organs of the garlic plant (Table 4). Isoforms of OASS varied significantly in their distribution in the garlic organs (Figure 5). Over-representation was notable in the cloves, roots, basal plate and leaves, whereas in the reproductive tissues, most of the annotated isoforms were less abundant. The isoforms were clustered into six groups, with clear expression patterns in the different organs. For instance, within cluster #3 (highlighted in light blue in Figure 5), the three variants were enriched exclusively in cloves. The 10 variants and isoforms of SAT were also differentially expressed in the six garlic organs (data not shown), but organ clustering differed from that of OASS.

**Table 4:** **Variants of nine enzymes involved in sulfur metabolism found in the garlic transcriptome**

**Enzyme name**	**Number of variants**
Ferredoxin-sulfite reductase	2
ATP sulfurylase	2
Cysteine synthase	27
Serine acetyltransferase	10
Glutamate–cysteine ligase	5
Glutathione synthetase	4
Adenosine 5'-phosphosulfate reductase	8
O-acetylserine(thiol)-lyase	26
Gamma-glutamyl transferase	22

Numerous genes have been reported in the organosulfur biosynthesis pathways in garlic and onion. These include ATP sulfurylase, sulfite reductase, CS, SAT, glutamate cysteine ligase and glutathione synthase from garlic [27], and ferredoxin-sulfite reductase (SiR), adenosine 5’-phosphosulfate reductase (APSR), OASS, glutamylcysteine synthase, glutathione synthase, and γ-glutamyl transpeptidase from the onion bulb [52,53]. Our transcriptome analysis suggests that in addition to a large repertoire of enzyme isoforms, the pathways of sulfur metabolism and translocation of organosulfur compounds between garlic organs are diverse: the cysteine sulfoxides are synthesized primarily in the leaves and roots, and are then translocated to the developing cloves. Further and more intensive studies combining biochemistry and genetic regulation of these pathways may improve our understanding of alliin production and translocation in garlic and probably other *Allium* plants.

### GO classification of garlic viruses

**Figure 6:**
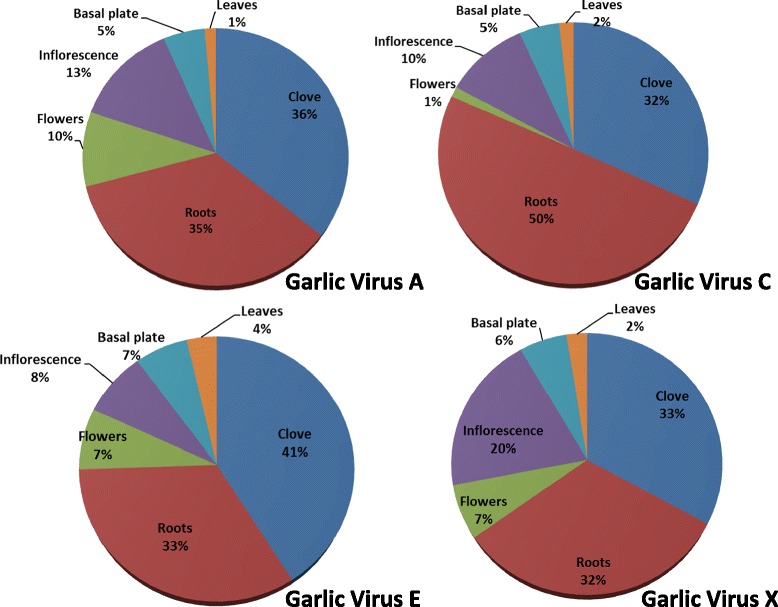
**Differential expression of four allexiviruses among six organs of garlic.** Note the highest proportion of virus expression in roots and cloves.

Garlic crops suffer heavy losses due to viral infections and other pests, and elimination of these viruses is particularly difficult. Moreover, due to the standard vegetative propagation of this crop, viruses are transmitted from one generation to the next [54]. The present transcriptome analysis was not designed to specifically search for garlic viruses. However, gene annotation revealed the presence of at least four known garlic allexiviruses: A, C, E and X. Interestingly, we were unable to find transcripts similar to the most common and agriculturally significant potyviruses *leek yellow stripe virus* (LYSV) and *onion yellow dwarf virus* (OYDV) (Table 5). In onion, *iris yellow spot tospovirus* (IYSV) is not transmitted by seeds [55], but it is not known whether potyviruses of garlic are. Our experimental genotype #87 was propagated and grown from a single seed in an insect-proof screenhouse, isolated from garlic and onion fields. Hence it might have been free of these viruses due to absence of inoculation. On the other hand, rather high amounts of the allexiviruses were identified in all tested organs, probably due to transmission via seeds. The highest amounts of viral RNA were identified in the roots and cloves. In contrast, flowers expressed only 1–10% of the total viral RNA amount, and the lowest number of reads (1–4%) was found in the leaves (Figure 6). This new information on the distribution of allexiviruses among the various plant organs provides a solid basis for future research and development of effective detection methods. In practice, leaves are often sampled for viral infection. It is evident that, at least for garlic allexiviruses, such sampling might result in erroneous estimations of viral infection, due to the relatively low titer in this organ.

**Table 5:** **List of known viruses annotated in garlic transcriptome**

**Virus**	**Total read count**
*Garlic virus C*	30,239
*Garlic virus A*	109,591
*Garlic virus E*	21,460
*Garlic virus X*	19,161
*Garlic latent virus*	0
*Leek yellow stripe virus*	0
*Onion yellow dwarf virus*	0
*Turnip mosaic virus*	0

Relatively high expression of the allexiviruses A, C, E and X was found, while no sequence matches to garlic potyviruses were annotated.

Our initial results suggest that transcriptome tools provide more sensitive detection methods for new and known viruses in garlic, and shed new light on plant–pathogen interactions and virus distribution in plant tissues.

## Conclusions

*De-novo* assembly of the transcriptome for fertile garlic provides a powerful tool for studying physiological, biochemical and genetic processes in garlic and other *Allium* crops. The first organ-specific transcript catalogue was generated herein using relatively long reads (MiSeq 250), based on six labeled libraries from vegetative and reproductive tissues. We created two catalogues: (1) an extensive transcriptome consisting of 240,000 sequences, and (2) an abundant transcriptome of 102,000 contigs, which were annotated and analyzed for GO and metabolic pathways. These datasets provide versatile resources for garlic genome research, and can be used to associate the transcriptome to developmental processes, understand the regulatory network of these processes, trace the expression profiles of individual genes, and identify reference genes for quantitative expression analyses in various organs and tissues. This tool is especially important in the elucidation of mechanisms of floral induction, micro- and megasporogenesis, male sterility and seed production in garlic. It will also be useful in the development of new methods for virus detection, as well as in the study of plant–pathogen interactions.

In the future, garlic “omics” studies will facilitate the development of user-friendly, efficient, transferable, and co-dominant markers such as SNPs, to be applied in current and future breeding programs. Molecular assisted breeding applications will be used in the selection of genotypes with desired traits, produced from true seeds.

## Methods

### Plant material

Fertile garlic clone #87 was derived from a single seed produced in 2004; further vegetative propagation guaranteed clonal uniformity. The plants were grown and propagated at the ARO, The Volcani Center, Bet Dagan, Israel. Freshly harvested bulbs were cured and stored under ambient conditions from July to September 2012 in an open shed. Following visual inspection, healthy looking propagules were transferred and stored under controlled conditions at 4°C and a RH of 65–70% for 8 weeks. In November, healthy cloves were disinfected and planted in a net-house in a medium consisting of 50:25:25 (v/v/v) volcanic tuff particles, compost, and ground coconut peel. The plants were fertigated regularly using “Shefer” liquid fertilizer (N:P:K = 59:35:94 g L-^1^, Dshanim, Israel)*.*

**Figure 7:**
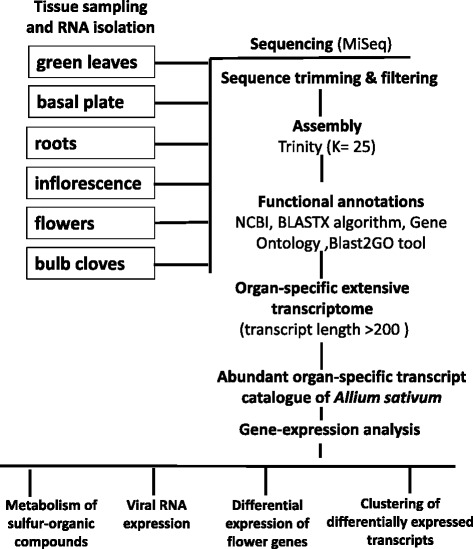
**Experimental design of the sequencing, assembly, annotation, construction and analyses of the organ-specific transcriptome catalogues of** ***Allium sativum.***

**Figure 8:**
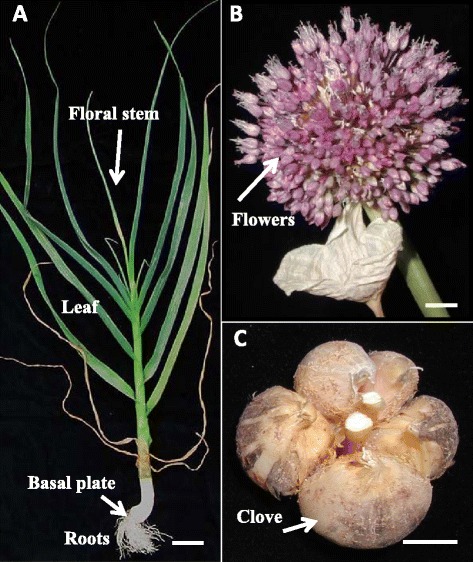
**Organography of tissue sampling in fertile garlic genotype #87.** Samples were collected in March–July 2013. **(A)** Image of the entire plant at vegetation stage, prior to flowering; bar = 7 cm. **(B)** Fully developed multi-flower inflorescence; bar = 0.8 cm. **(C)** Mature bulb consisting of a few cloves; bar = 1 cm.

The experimental design is presented in Figure 7. Tissue samples were collected in three biological replicates between March and July 2013 from (1) the bulb basal plate; (2) green foliage leaves and scape; (3) roots; (4) young inflorescences with developing flower primordia, spathe removed; (5) individual flowers at various stages of development, and (6) cloves (Figure 8). The freshly sampled tissues were immediately dipped in liquid nitrogen and stored at -80°C until RNA extraction.

### RNA isolation and sequencing procedures

Total RNA was isolated from flowers and inflorescences using RNeasy Mini Kit (Qiagen, Hilden, Germany) according to the manufacturer’s instructions. For the vegetative tissues, the CTAB protocol was used [56]. RNA extract quality was verified using the Agilent 2100 Bioanalyzer with a minimum RNA integrated number value of 7.

Total RNA samples were shipped to the Roy J. Carver Biotechnology Center, W.M. Keck Center for Comparative and Functional Genomics, Urbana, IL, USA for library preparation and sequencing. The RNAseq libraries were prepared with Illumina's TruSeq RNA Sample Prep Kit (www.Illumina.com) with one modification: RNA was randomly primed but not chemically fragmented. The pool was quantified by qPCR and sequenced for 251 cycles from each end of the fragments on a MiSeq v3 platform using a MiSeq sequencing kit, version 3, with a relatively long read length of 250 nt, and analyzed with Casava 1.8 (pipeline 1.8).

### *De novo* sequence assembly

Raw reads were subjected to a cleaning procedure using the FASTX Toolkit (http://hannonlab.cshl.edu/fastx_toolkit/index.html, version 0.0.13.2) including: (1) trimming read-end nucleotides with quality scores < 30 using fastq_quality_trimmer; (2) removing read pairs if either one had less than 70% base pairs with quality score ≤ 30 using fastq_quality_filter. A total of 28.4 million cleaned paired-end reads, obtained after processing and cleaning, were assembled *de novo* using Trinity software (version trinityrnaseq_r2013_08_14 [57] with default parameters and 25mer k-mer size. For assembly, the initial parameters of Trinity were set as follows: --seqType fq --JM 400G --CPU 10 --bflyHeapSpaceMax 400G --bflyHeapSpaceInit 400G --bflyCPU 10. The assembled sequences that shared a number of k-mers (the set of isoforms of a gene) were referred to as “contigs”. The sets of all sequences that shared at least one k-mer were referred to as components.

The resulting *de novo* assembly was used to generate two transcriptomes: the “extensive transcriptome” and the “abundant transcriptome”. The extensive transcriptome consisted of all of the contigs, whereas the abundant transcriptome included only the contigs with count per sample ≥10. CD-HIT (http://www.bioinformatics.org/cd-hit/) with 95% global sequence identity was used to cluster the sequences of both transcriptomes.

For the abundant transcriptome compilation, filtration of the likely contig artifacts and low expressed contigs was carried out as follows: (1) abundance estimates were calculated for each contig using the RSEM software package [58]; (2) only contigs representing more than 1% of the per-component (IsoPct) expression level were retained; (3) finally, contigs with total mapped reads of less than 10 were removed.

### Data availability

The sequencing data were deposited in the NCBI Sequence Read Archive (SRA) database as bioproject PRJNA243415 [GenBank:SRR1219535, GenBank:SRR1219644, GenBank:SRR1219646, GenBank:SRR1219796, GenBank:SRR1219989, GenBank:SRR1220207].

### Sequence similarity and functional annotation

To assess the similarity of the garlic transcriptome to those of other model and closely related species, analysis of sequence similarity was performed using the BLAST (Basic Local Alignment Search Tool) algorithm with an E-value cut-off of 10^−5^ [59].

Comparison to the transcriptomes of the garlic renewal bud [27], onion [26] and *Tulipa* and *Lilium* [24,25] was performed using the BLASTN algorithm, which allows comparison using a nucleotide query. In addition, the BLASTX algorithm was used to search protein databases using a translated nucleotide query for the comparison of the assembled contigs with sequences deposited in the databases of *Arabidopsis* (http://www.arabidopsis.org), *Oryza sativa* (www.phytozome.org) and NCBI non-redundant (nr) proteins (http://www.ncbi.nlm.nih.gov; http://www.ncbi.nlm.nih.gov/genbank/statistics). Blosum62 matrix was used for BLASTX (when searching against a protein database with a DNA query), TBLASTN (when searching for translated nucleotide databases with a protein query) and TBLASTX (when searching for translated nucleotide databases with a translated nucleotide query). BLASTN was executed using the default parameters of the Blastall program (version 2.2.25).

GO annotations were assigned using Blast2GO [60] based on an nrNCBI search. GO provides a structured and controlled terminology to describe the gene products, according to three categories: molecular function (refers to the biochemical activity of a gene product without stating the location of the event), biological process (refers to the biological objective to which the gene product contributes), and cell component (refers to the place in the cell where a gene product is active). GO-enrichment analysis was performed using Fisher's Exact Test of Blast2GO software. Analysis was carried out by comparing the GO terms in the test sample to the GO terms in a background reference. A false discovery rate [61] with corrected P-value of less than 0.05 was set as the threshold. The REViGO web server was used for visualization of the GO terms in a semantic similarity-based scatterplot (http://revigo.irb.hr/ [62]).

In the search for orthologous genes involved in flowering induction or sulfur metabolism, 22 candidate sequences were retrieved from databases in the NCBI (Additional file 4: Table S2). Sequences were compared with the abundant garlic transcriptome using TBLASTN, with an E-value cut-off of 10^−5^.

### Abundance estimation and differential expression analysis

The cleaned reads from each organ were aligned separately with the abundant transcriptome assembly using the Bowtie aligner [63]. Following the Trinity protocol [64], the abundance estimation was calculated using the run_RSEM_align_n_estimate.pl script Perl. Since reads are generally shorter than transcripts from which they are derived, a single read may map to multiple genes and isoforms, thus complicating expression analyses. Therefore, the transcript quantification from RNA-Seq data was performed using the Expectation-Maximization method (RSEM), which handles read mapping uncertainty with a statistical model by estimating maximum likelihood expression levels [58,65]. Bioconductor EdgeR package [66] in the R environment was used to identify differentially expressed transcripts for each pair of samples (15 comparisons overall), based on the count estimations for each contig. Transcripts that were more than fourfold differentially expressed with false discovery- corrected statistical significance of at most 0.001 were considered differentially expressed.

### Cluster analysis

The expression patterns of the transcripts in different organs were studied using cluster analysis of the differentially expressed transcripts in at least one pairwise sample comparison. Following the Trinity protocol [64], expression normalization was calculated using TMM (trimmed mean of M-values), following FPKM (fragments per feature kilobase per million reads mapped) calculations. Then, hierarchical clustering of transcripts and samples was performed and clusters were extracted using R scripts.

### Analysis of viral genome sequences

Eight genome sequences of garlic viruses were downloaded from the NCBI genome database (ftp://ftp.ncbi.nlm.nih.gov/genomes/Viruses/), including: *leek yellow stripe virus* (LYSV) (NC_004011.1), *onion yellow dwarf virus* (OYDV) (NC_005029.1), *garlic virus C* (NC_003376.1), *garlic virus A* (NC_003375.1), *garlic virus E* (NC_004012.1), *garlic virus X* (NC_001800.1), *garlic latent virus* (NC_003557.1) and *turnip mosaic virus* (TMV) (NC_002509.2). The cleaned reads, obtained separately from each organ, were aligned independently to the eight viruses using Bowtie2 aligner (version 2.1.0; [67]) and the counts were estimated using the SAM tools software [68].

## Additional files

Additional file 1: Figure S1. Representation of enriched biological processes and functions in clusters #1 and 2 (Figure 3), as analyzed by Blast2GO and REViGO tools. The biological process terms are arranged in semantic space and colored by semantic positioning on the X-axis, the size of bullet points by significance (log10 of P-value). Only GO terms with contig counts higher than 1% are shown. (a) Enriched biological processes and molecular functions in cluster #1, predominantly in the basal plate and roots. The main processes include responses to various types of stress and metabolite transport. (b) Enriched biological processes and molecular functions in cluster #2, predominantly in the basal plate. The main processes include catalytic activity and interaction with virus. Additional file 2: Figure S2. Representation of enriched biological processes and functions in clusters #3 and 4 (Figure 3), as analyzed by Blast2GO and REViGO tools. The biological process terms are arranged in semantic space and colored by semantic positioning on the X-axis, size of bullet points by significance (log10 of P-value). Only GO terms with contig counts higher than 1% are shown. (a) Enriched biological processes and molecular functions in cluster #3, mainly in the reproductive organs. The two main groups include the pathways of florogenesis/gametogenesis and DNA replication/modification. (b) Enriched biological processes and molecular functions in cluster #4, mainly in the foliage leaves. The main processes include response to light quality and intensity, photosynthesis, and biosynthesis of the secondary metabolites. Additional file 3: Table S1. List of the 12 most abundant proteins in the vegetative and reproductive organs of garlic. The expression level (FPKM) was calculated via the expectation maximization method and TMM normalization (trimmed mean of M-value normalization method) was applied (see Materials and Methods). The annotation was based on the comparison with nrNCBI using BLASTX. Note over-representation of three non-annotated proteins in leaves and flowers. Additional file 4: Table S2. Candidate sequences of genes involved in flowering and sulfur metabolism, retrieved from NCBI databases (April 2014).
