# Supplementary figures and images for: Integrated transcriptome catalogue and organ-specific profiling of gene expression in fertile garlic (Allium sativum L.)
Source: BMC Genomics. 2015 Jan 22;16(1):12. doi: 10.1186/s12864-015-1212-2 (PMC4307630; doi:10.1186/s12864-015-1212-2)

## Slide 1
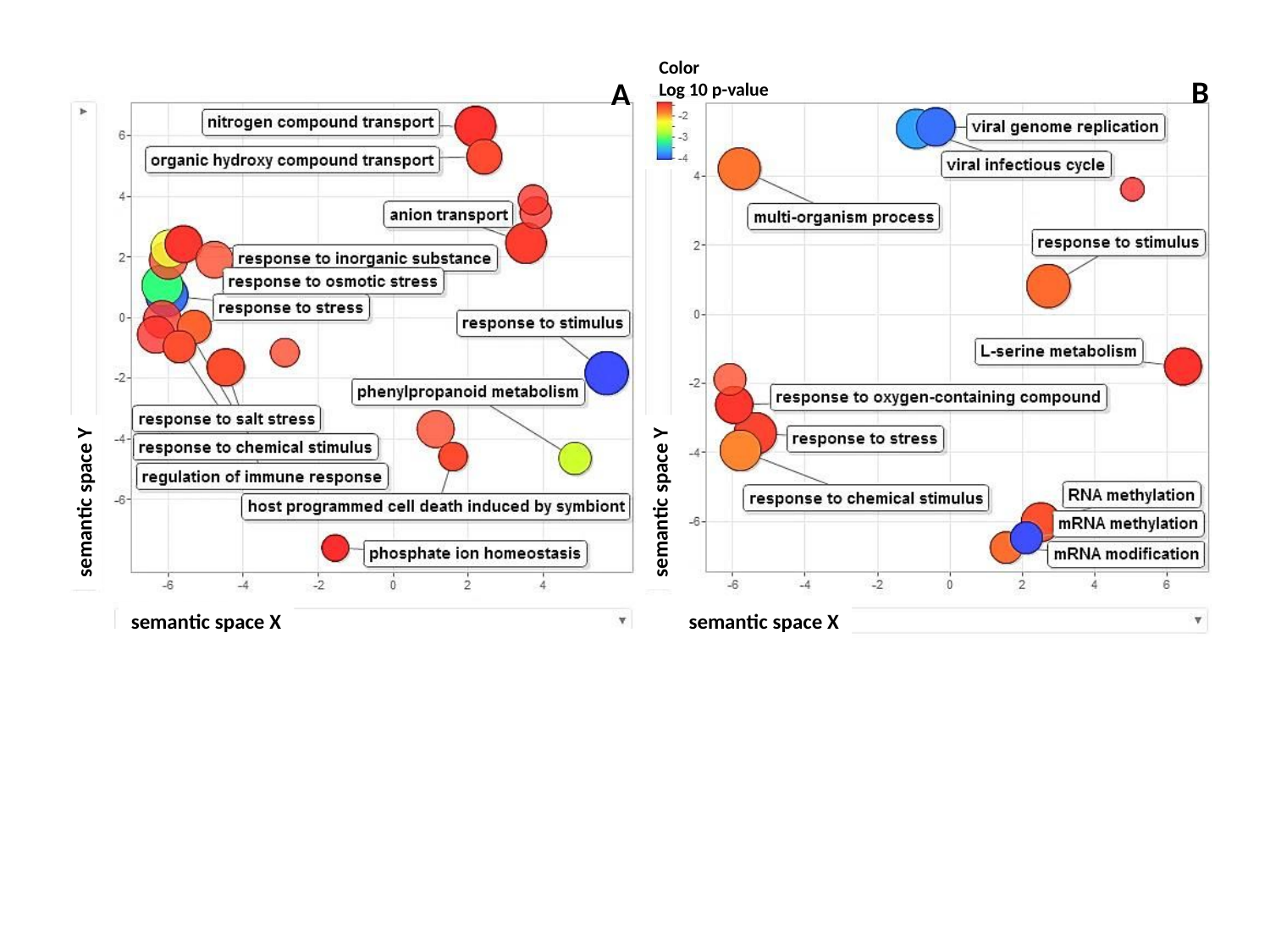

Color
Log 10 p-value
B
A
semantic space Y
semantic space Y
semantic space X
semantic space X

Supplement: Additional file 1: Figure S1. — Representation of enriched biological processes and functions in clusters #1 and 2 (Figure 3), as analyzed by Blast2GO and REViGO tools. The biological process terms are arranged in semantic space and colored by semantic positioning on the X-axis, the size of bullet points by significance (log10 of P-value). Only GO terms with contig counts higher than 1% are shown. (a) Enriched biological processes and molecular functions in cluster #1, predominantly in the basal plate and roots. The main processes include responses to various types of stress and metabolite transport. (b) Enriched biological processes and molecular functions in cluster #2, predominantly in the basal plate. The main processes include catalytic activity and interaction with virus. [file 12864_2015_1212_MOESM1_ESM.pptx]

## Slide 1
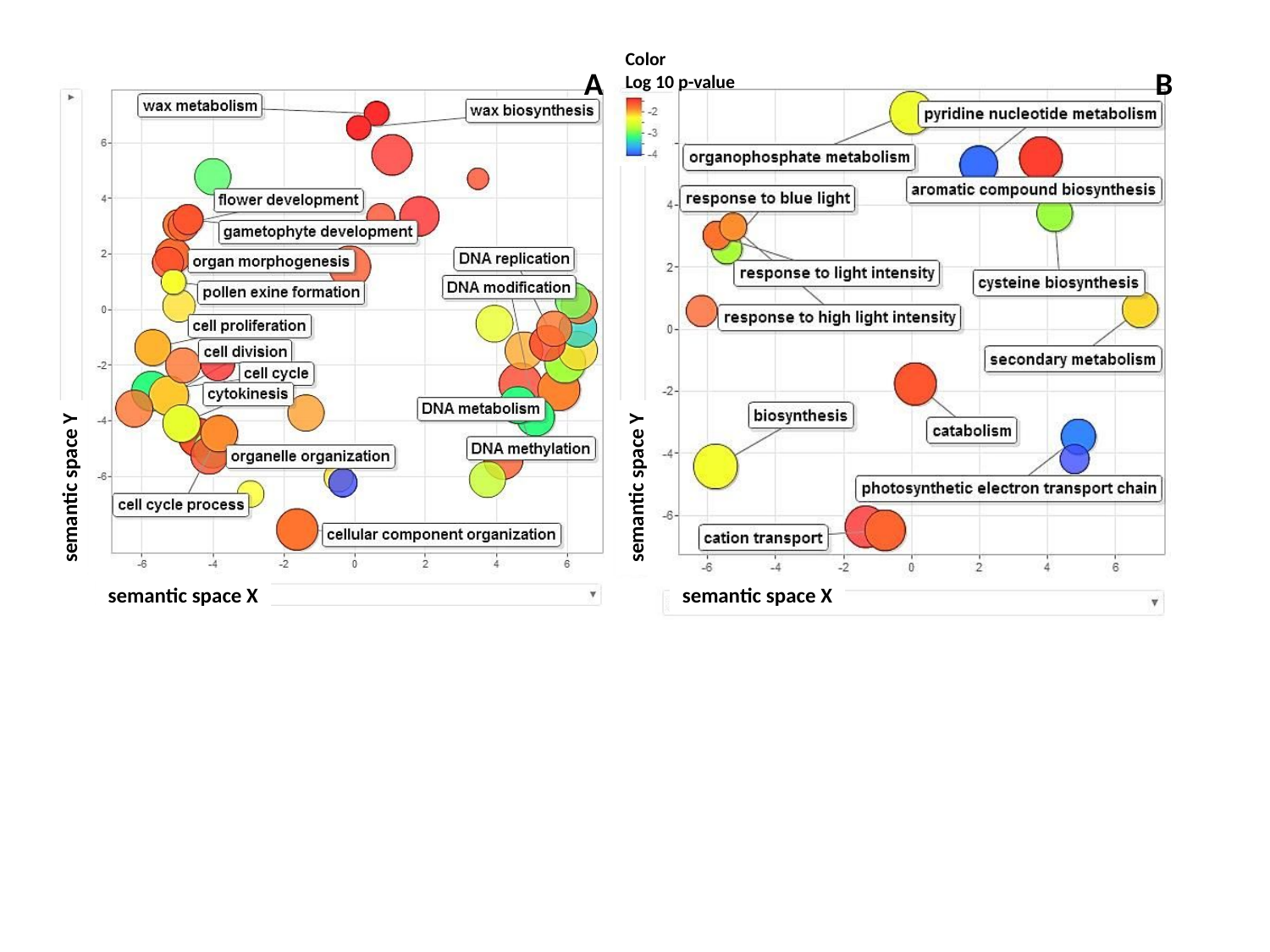

Color
Log 10 p-value
A
B
semantic space Y
semantic space Y
semantic space X
semantic space X

Supplement: Additional file 2: Figure S2. — Representation of enriched biological processes and functions in clusters #3 and 4 (Figure 3), as analyzed by Blast2GO and REViGO tools. The biological process terms are arranged in semantic space and colored by semantic positioning on the X-axis, size of bullet points by significance (log10 of P-value). Only GO terms with contig counts higher than 1% are shown. (a) Enriched biological processes and molecular functions in cluster #3, mainly in the reproductive organs. The two main groups include the pathways of florogenesis/gametogenesis and DNA replication/modification. (b) Enriched biological processes and molecular functions in cluster #4, mainly in the foliage leaves. The main processes include response to light quality and intensity, photosynthesis, and biosynthesis of the secondary metabolites. [file 12864_2015_1212_MOESM2_ESM.pptx]
