# Supplementary material for: Integrated transcriptome catalogue and organ-specific profiling of gene expression in fertile garlic (Allium sativum L.)
Source: BMC Genomics. 2015 Jan 22;16(1):12. doi: 10.1186/s12864-015-1212-2 (PMC4307630; doi:10.1186/s12864-015-1212-2)
Supplement: Additional file 3: Table S1. — List of the 12 most abundant proteins in the vegetative and reproductive organs of garlic. The expression level (FPKM) was calculated via the expectation maximization method and TMM normalization (trimmed mean of M-value normalization method) was applied (see Materials and Methods). The annotation was based on the comparison with nrNCBI using BLASTX. Note over-representation of three non-annotated proteins in leaves and flowers. [file 12864_2015_1212_MOESM3_ESM.pdf]

Additional Table 1 List of the 12 most abundant proteins in the vegetative and reproductive organs of garlic. The expression level (FPKM) was calculated via the expectation maximization method and TMM normalization (trimmed mean of M-value normalization method) was applied (see Materials and Methods). The annotation was based on the comparison with nrNCBI using BLASTX. Note over-representation of three non-annotated proteins in leaves and flowers

	Protein homology	Roots	Basal Plate	Cloves	Leaves	Inflorescence	Flowers
1	cytochrome P450- like TBP [*Medicago truncatula*]	11123.7	23381.5	12434.2	*34911.8	7688.1	4255.6
2	hypothetical protein MTR_5g051000 [*Medicago truncatula*]	*5873.5	4887.6	4623.5	4301.4	5084.3	2767.3
3	alliinase-1 [*Allium sativum*]	1.8	503.2	*4776.4	4406.8	2164.6	2576.6
4	non-annotated	44759.8	50142.1	16173.6	*51437.9	4178.1	2404.4
5	non-annotated	5464.4	9289.3	7227.7	*20981.9	398.2	528.1
6	lectin I, partial [*Allium sativum*]	5.9	65.2	*7033.2	0	0	0
7	tau glutathione S-transferase [*Allium cepa*]	1010.2	727.1	*6292.2	1053.4	658.5	428.8
8	metallothionein type 2 [*Allium sativum*]	968.7	*21929.2	230.7	1666.3	651.5	382
9	trypsin inhibitor [*Allium cepa*]	69.2	754.9	1442.6	*11154.1	891.6	548.2
10	S-adenosylmethionine synthase 2 [*Vitis vinifera*]	*4802.8	3411.1	1794.6	3533.9	1876.6	1107.5
11	cationic peroxidase [*Nelumbo nucifera*]	2436.1	2572.6	703.3	2277.7	*3159.9	1239.5
12	non-annotated	0	0	1.8	1.5	0	*4258.9

*****The highest expression level in the specific organ.
