# Supplementary material for: Integrated transcriptome catalogue and organ-specific profiling of gene expression in fertile garlic (Allium sativum L.)
Source: BMC Genomics. 2015 Jan 22;16(1):12. doi: 10.1186/s12864-015-1212-2 (PMC4307630; doi:10.1186/s12864-015-1212-2)
Supplement: Additional file 4: Table S2. — Candidate sequences of genes involved in flowering and sulfur metabolism, retrieved from NCBI databases (April 2014). [file 12864_2015_1212_MOESM4_ESM.pdf]

Additional Table 2 Candidate sequences of genes involved in flowering and sulfur metabolism, retrieved from NCBI databases (April 2014)

| Gene name | Number in NCBI database | Reference species |
|---|---|---|
| *FLOWERING LOCUS T (FT)* | JX145040.1 | *Allium cepa* |
| *CONSTANS (CO)* | KC677633.1 | *Allium cepa* |
| *SUPPRESSOR OF OVEREXPRESSION OF CONSTANS1* (*SOC1*) | KC677637.1 | *Allium cepa* |
| *LEAFY (LFY)* | AY563105.1 AY672745.1 | *Allium sativum* |
| *APETALA1 (AP1* | KF170023.1 | *Allium cepa* |
| *APETALA2 (AP2)* | XM_006658355.1 | *Oryza brachyantha* |
| *APETALA3 (AP3)* | JX661502.1 | *Allium cepa* |
| *PISTILLATA* (*PI)* | DQ005594.1 | *Allium ursinum* |
| *PISTILLATA* (*PI*)] | JX679083.1 | *Allium cepa* |
| *SEPALLATA1* (*SEP1*) | KF673860.1 | *Phalaenopsis equestris* |
| *SEPALLATA3* (*SEP3*) | KF170022.1 | *Allium cepa* |
| *AGAMOUS* (*AG*) | KF170020.1 | *Allium cepa* |
| Ferredoxin-sulfite reductase | AAU90245.1 | *Oryza sativa* Japonica Group |
| ATP sulfurylase | AAP13004.1 | *Oryza sativa* Japonica Group |
| Cysteine synthase | AAK71541.1 | *Oryza sativa* Japonica Group |
| Serine acetyltransferase | Q8W0E4.1 | *Oryza sativa* Japonica Group |
| Glutamate--cysteine ligase | Q8GU95.2 | *Oryza sativa* Japonica Group |
| Glutathione synthetase | AAS19531.1 | *Oryza sativa* Japonica Group |
| Adenosine 5'-phosphosulfate reductase | ABA99457.1 | *Oryza sativa* Japonica Group |
| O-acetylserine(thiol)-lyase | BAD82695.1 | *Oryza sativa* Japonica Group |
| Gamma-gluttonyl cysteinyl transferase | NP_001052974.2 | *Oryza sativa* Japonica Group |
| Gamma-glutamyl transferase | AAL61611.2 | *Allium cepa* |
